# On the efficacy of preventive toltrazuril treatments and the diagnosis of *Cystoisospora suis* infections in intensively raised piglets in farms from southeast Spain

**DOI:** 10.1007/s00436-024-08127-y

**Published:** 2024-01-24

**Authors:** Georgiana Deak, Lola González-Amador, Elena Goyena, Andrada-Silvia Cârstolovean, José Risueño, Eduardo Berriatua

**Affiliations:** 1 Department of Parasitology and Parasitic Diseases, University of Agricultural Sciences and Veterinary Medicine of Cluj-Napoca, Calea Mănăștur 3-5, 400372 Cluj-Napoca, Romania; 2 Department of Animal Health, Faculty of Veterinary Science, Regional Campus of International Excellence ‘Campus Mare Nostrum’, University of Murcia, 30100 Murcia, Spain

**Keywords:** Cystoisosporosis, Neonatal coccidiosis, Diagnosis, Pigs, Resistance

## Abstract

This study investigated the presence and abundance of *Cystoisospora suis* oocysts in faecal samples from 131 one- to three-week-old pig litters belonging to eight intensively raised, indoor herds in Spain. Seven herds used preventive anticoccidial toltrazuril treatments administered orally or by intramuscular injection, and one did not use preventive anticoccidial treatments. The diagnosis was performed using two oocyst flotation-concentration methods, Bailenger’s method in every herd and the more recent Joachim’s method in four herds. Oocysts were detected in every farm, the proportion of oocyst-positive samples was higher with Bailenger’s technique, and the estimated overall prevalence (95% confidence interval) was 40 (32–49)%, including 47 (29–65)% in non-medicated litters, 52 (38–67)% in orally medicated litters and 28 (16–40)% in intramuscularly medicated litters (*p* < 0.05). However, mixed logistic regression models indicated that the risk of infection was not significantly associated with preventive anticoccidial treatments (*p* > 0.05), and increased with age, was higher in herds with partially compared to fully slatted dung floors in farrowing pens and in litters with pigs with diarrhoea (*p* < 0.05). The median (range) oocysts per gram of faeces (OpG) in infected litters by Bailenger’s method was 623 (35–49048) and mixed negative binomial models revealed no significant association between infection intensity in positive litters and pen’s floor type and piglets age, faecal consistency and treatment status (*p* > 0.05). The apparent low efficacy of Toltrazuril suggests treatment administration failures, reduced residual efficacy or low susceptibility of *C*. *suis* strains in study farms and needs further investigation.

**Supplementary Information:**

The online version contains supplementary material available at 10.1007/s00436-024-08127-y.

## Introduction

*Cystoisospora suis* (previously named *Isospora suis*) is an apicomplexan protozoan parasite causing neonatal coccidiosis in piglets, an intestinal disease that is highly prevalent in intensive farming systems (Joachim and Shrestha 2020). Piglets become infected shortly after birth through the ingestion of sporulated oocysts in the environment from previously infected litters, as dams are considered a minor source of infection to their offspring (Lindsay et al. 1997; Sotiraki et al. 2008). The parasite targets mainly jejunum and ileal enterocytes, and following a short 5-to-6-day prepatent period, large numbers of unsporulated oocyst are eliminated in the faeces which, under the warm, humid conditions of farrowing units, sporulate in <12–48 h (Ernst et al. 1986; Lindsay et al. 1997). Most commonly affected animals are 2 to 3 weeks old, subclinical infection is associated with reduced weight gains and typical clinical signs include non-hemorrhagic diarrhoea, dehydration, lethargy, body weight loss and death (Maes et al. 2007; Hinney et al. 2020).

Correct husbandry and hygienic practices that reduce oocyst accumulation in the environment and piglet exposure to sporulated oocysts are key to disease prevention (Straberg and Daugschies 2007; Sotiraki et al. 2008). Still, the efficacy of management interventions in preventing losses associated with infection is limited and generally, additional chemoprophylactic treatment of piglets with anticoccidials is commonly used (Hinney et al. 2020; Hinney et al. 2021). The ample, sustained use of anticoccidials for the control of coccidiosis in intensively raised farm animals has led to long-time encountered resistance against these products (Noack et al. 2019). Among them, toltrazuril (TZ) is a triazinetrione derivative that interferes with the nuclear division of asexual (merozoite) and sexual (gametocyte) coccidial stages. It is widely used for the prevention of coccidiosis in piglets and is the only preventive treatment licensed in the European Union (EMA 2000; Shrestha et al. 2017; Gong et al. 2021; Karembe et al. 2021). It is typically administered to piglets in the first week of life by oral dosing (EMA 2000) and more recently, an intramuscular injection combining toltrazuril and gleptoferron (Iron-III) is also available, providing a higher and more sustained tissue and faecal concentration of the active compound (Karembe et al. 2021). However, there is already a report of a *C*. *suis* field isolate from a Dutch farm developing resistance to TZ, evidenced by similar oocysts excretion, faecal consistency and body weight gain in treated and untreated pigs experimentally infected with this isolate (Shrestha et al. 2017). The authors highlighted the need for developing experimental testing methods to facilitate the detection of inevitable further *C*. *suis* TZ-resistant isolates.

Parasitological diagnosis in the live animal relies on oocyst identification and quantification, commonly done using coprological parasite flotation-concentration methods and McMaster counting chambers which require minimal laboratory infrastructure (Taylor et al. 1995; Gong et al. 2021). Such methods rely on oocysts floating and concentrating on the top layer of a density gradient formed in a test tube containing a filtrate of faeces homogenized in a high-density salt or sugar solution. However, the high concentration of milk fat in piglets’ faecal samples is problematic. Fat concentrates on top of the flotation medium enclosing and impeding many oocysts from reaching the top cover of the McMaster chamber and they cannot be visualized under light microscopy. In such circumstances, the number of oocysts per g of faeces (OpG) is grossly underestimated (Joachim et al. 2018b). In an attempt to overcome this limitation, Joachim et al. (2018a) adapted the gauze-filtration method described by Henriksen and Christensen (1992). It is based on filtering the faeces-high-density homogenate solution by pushing down a double-layered piece of gauze attached to a metal loop through the solution to the bottom of the test tube, thereby preventing unsuspended fat droplets from reaching the surface and trapping the oocysts. The method, used for individual pig faeces, proved to be less sensitive than faecal smears used for autofluorescence but was not compared to standard flotation concentration methods (Joachim et al. 2018a).

Spain is now the largest pig producer in the European Union (MAPA, 2021), yet there is limited information on the epidemiology and control of *C*. *suis* infections (Hiob et al. 2019; Hinney et al. 2020). In the present study, we investigated oocyst excretion in piglets aged 1 to 3 weeks old from eight farms in Murcia Region, in southeast Spain, seven of which used TZ preventively administered orally or by intramuscular injection and one did not use anticoccidials. We used mixed multivariable modelling to analyze the relationship between the presence and abundance of oocysts in faeces and the use or not of TZ preventive medication and other potential risk factors of infection. Also, we compared the diagnostic performance of the gauze-filtration-flotation method described by Joachim et al. (2018a) with a classical modified Bailenger’s flotation-concentration method (Ayres and Mara 1996).

## Materials and methods

### Study design and population

The present observational, cross-sectional study of *C*. *suis* infection in piglets was carried out in Murcia Region, a relatively small region in southeast Spain (1°7′48.14″ W; 37°59′13.34″ N; 11,313 km^2^ and 1.5 million inhabitants), which in 2021 held 1.7% of the pig farms (1433 farms) and 7.2% of the pigs (2.8 million pigs) in Spain (MAPA 2020). The study was done in eight indoor, intensive, farrow-to-finish and farrow-to-feeder production farms, with a census ranging between 280 and 3700 breeding sows. They were conveniently selected, representing all the clients under the responsibility of a veterinary practitioner (EG), and none reported clinical cystoisosporosis in their farms for more than 1 year at the time of selection, characterized by a high proportion of diarrhoeic piglets, confirmed by laboratory detection of large number of oocysts/g in the faeces of affected animals and absence of other common neonatal pathogens.

Faeces samples were collected between 2019 and 2023, from 655 randomly selected one- to three-week-old piglets born to 131 similarly selected sows. This corresponded to 5 piglets/litter, a sample size large enough to detect infection with a 95% probability if at least 50% of the piglets shed oocysts (Thrusfield 2007) and considering an average of 12.1 piglets/litter in Spain (López 2019). Litters and piglets were randomly selected and *C*. *suis* analysis were performed at litter-level as described below. Except for one farm (farm 5), the remaining farms used TZ anticoccidial preventive treatments when piglets were 48–72 h old. They included orally administered Expacox 5% (Livisto) in 3 farms (farms 2, 3 and 4) and intramuscularly injected Forceris® (CEVA) in the other 4 farms (farms 1, 6, 7 and 8). Treatments were administered at the recommended doses of 20 mg/kg of Espacox and 45 mg/piglet for Forceris®. Pigs were kept in farrowing pens with heated plates, and dung areas were fully slatted in six farms and partially slatted in two farms (farms 2 and 4).

When the study started in 2019, four farms (farms 4, 5, 6 and 8) were tested for *C*. *suis* infection by Bailenger’s diagnostic method. When the study restarted in 2022, after being allowed access to farms following the peak of the COVID-19 pandemic, the investigation was completed by including a further four farms (1, 2, 3 and 7). Moreover, samples from these four farms were tested using Bailenger’s and Joachim’s diagnostic methods in an attempt to provide some information on their ability to detect litter infection and estimate oocyst excretion rates. All farms were sampled in winter, between November and February.

### Faecal sampling and consistency assessment

Litter samples were collected individually from the rectum of piglets using individual cotton swabs introduced in the rectum to stimulate defecation, and samples from the same litter were placed in individual 150 ml sterile plastic containers. They were transported to the laboratory in isothermal boxes with ice blocks and kept refrigerated until analyzed within 5 days of collection. The consistency of faeces was assessed and classified as firm, pasty, semi-liquid or liquid, with the latter two rated as diarrhoeic (Joachim et al. 2014). The faecal consistency with the lowest density (liquid<semi-liquid<firm) of the five samples in the litter was recorded as the litter’s faecal status and used for statistical analysis of its relationship with oocyst excretion as described below. Samples from the same litter were then thoroughly mixed together with a spatula in the container where they were collected, to form a single homogeneous sample for parasitological analysis.

### Parasitological analysis

Samples were analyzed with one or two diagnostic methods: Joachim et al. (2018a) (Joachim’s method hereafter) using 0.5 g of faeces and saccharose-saturated flotation solution, and Bailenger’s method (Ayres and Mara 1996) with some modifications (supplementary digital video of the procedures is provided). Specifically, 1.5 g of faeces were mixed with 20 ml of acetoacetic buffer (15 g sodium acetate anhydrous, 6 g acetic acid glacial, 1 l H_2_O) with a pestle and mortar for 5 min. The mixture was then filtered through a double-layered piece of gauze laid over a tea strainer into a 150 ml plastic container. A 5 ml aliquot of the filtrate was transferred to a 12 ml glass test tube for quantitative parasitological analysis using a McMaster counting chamber. Following, 5 ml of Petroleum ether was added to the tube containing the filtrate aliquot, and the contents were thoroughly shaken by hand to form a uniform mixture that was centrifuged at 1000 g for 10 min. After centrifugation, four distinct layers were recognized from top to bottom: ether, fatty ring, acetoacetic buffer and the sediment including oocysts (when present). After loosening the fatty ring from the sides of the tube using a narrow spatula, the tube was emptied leaving only the sediment which was resuspended with a vortex mixer. The saturated-saccharose solution was then added to the 5 ml mark and after homogenization, the solution was used to load both sides of a McMaster chamber which 5 min later was examined microscopically at ×200 magnification.

### Data analysis

Litters were considered positive when at least one oocyst was detected in the pooled litter sample, and litter infection intensity was defined as the number of OpG in positive litters. The following formula was employed to calculate the number of OpG in Joachim’s method (Joachim et al. 2018a):

$$OpG=X\times \frac{100}{VolEx\ ml\ }$$

where:

OpG: number of oocysts per gram of faeces

*X*: number of oocysts counted

VolEx ml: volume of flotation solution examined in milliliters, typically 0.15 ml.

Similarly, for Bailenger’s method (Ayres and Mara 1996), the number of OpG was calculated with the following formula:

$$\textrm{OpG}=\frac{\textrm{X}\times \left(\textrm{FWg}+\textrm{VolAAcB}\ \textrm{ml}\right)}{\textrm{VolEx}\ \textrm{ml}\times \textrm{FWg}}$$

where:

OpG: number of oocysts per gram of faeces

*X*: number of oocysts counted

FW g: faeces weight in grams

VolAAcB ml: volume of acetoacetic buffer in milliliters

VolEx ml: volume of flotation solution examined in milliliters.

The estimated litter prevalence of *C*. *suis* was the proportion of positive litters. Prevalence and median intensities across independent explanatory variables including litter age in weeks (one, two or three), floor type in the dung area of the farrowing pen (partially or totally made of slats allowing faeces to go through), faecal consistency (firm, pasty or diarrhoeic), preventive treatment administration (none, oral TZ or injected TZ), year (2019, 2022 or 2023) and farm (1 to 8) (Table 1) were analyzed using Pearson’s chi-square tests (and Fisher’s exact test when necessary) and the non-parametric Kruskal-Wallis rank sum test, respectively (Kirwood and Sterne 2003). Random effects generalized linear models were developed to explore the independent contribution of explanatory variables age, floor type, faecal consistency, treatment, year and fam and the interactions between treatment and age and floor types, to oocysts excretion (assessed with Bailenger’s method), specifically: (i) to the presence/absence of *C*. *suis* in litter faeces, by logistic regression and (ii) to oocysts counts (the number of oocysts/g) in oocyst-excreting litters, using Poisson and negative binomial regression. These distributions are typically used for count data i.e. obtained from counting and with non-negative values; however, the negative binomial distribution is preferred when overdispersion (variance exceeds the mean) is present. Explanatory variables we fitted in the models as fixed effects except for farm that was included as a random effect with variation considered at the intercept level, to account for sources of unobserved variation/heterogeneity at the herd level (Demidenko 2013). We selected a saturated model including every explanatory variable and excluding interaction terms if not significantly associated with the outcome, and the model was fit maximizing the likelihood.

**Table 1:** *Cystoisospora suis* prevalence and infection intensity in positive litters from eight farms, analyzed using Bailenger´s filtration-flotation concentration method

Variable	Level	No.	No. +ve.	Prevalence	*P* value	Oocysts/g faeces		*P* value
		litters	litters	(95%CL)		mean	median (range)	
Overall		131	53	40 (32-49)		5880	623 (35-49048)	
Farm	1	15	6	40 (15-65)	0.0288	5500	825 (37-19323)	0.0008
	2	15	9	60 (35-85)		2359	147 (73-18920)	
	3	15	4	27 (4-49)		46	37 (37-73)	
	4	14	10	71 (48-95)		7508	3192 (360-22961)	
	5	30	14	47 (29-65)		8025	799 (46-49048)	
	6	16	4	25 (0-46)		13757	9445 (3565-32574)	
	7	16	5	31 (9-54)		146	73 (35-330)	
	8	10	1	10 (0-29)		14026	14026 (14026-14026)	
Age (weeks)	1	43	8	19 (7-30)	0.0002	3387	1011 (35-18920)	0.0757
	2	56	23	41 (28-54)		9925	3526 (37-49048)	
	3	32	22	69 (53-85)		2558	338 (37-19323)	
Treatment	none	30	14	47 (29-65)	0.0358	8025	799 (46-49048)	0.8317
	oral toltrazuril	44	23	52 (38-67)		4195	477 (37-22961)	
	injectable toltrazuril	57	16	28 (16-40)		6424	825 (35-32574)	
Treatment; age	none; 1	12	1	8 (0-24)	0.0009	1911	1911 (1911-1911)	0.2904
	none; 2	8	5	63 (29-96)		17863	3526 (46-49048)	
	none; 3	10	8	80 (55-100)		2641	364 (47-10159)	
	oral; 1	13	4	31 (6-56)		5377	1240 (110-18920)	
	oral; 2	20	11	55 (33-77)		6585	2530 (37-22961)	
	oral; 3	11	8	73 (46-99)		318	129 (37-1224)	
	injectable; 1	18	3	17 (0-34)		1224	73 (35-3565)	
	injectable; 2	28	7	25 (9-41)		9502	6195 (147-32574)	
	injectable; 3	11	6	55 (25-84)		5433	679 (37-19323)	
Faecal consistency	firm	65	20	31 (20-42)	0.0173	3334	393 (35-21428)	0.4175
	pasty	33	13	39 (23-56)		9138	623 (37-49048)	
	diarhoeic	33	20	61 (44-77)		6307	1748 (46-35567)	
Floor type	completely slatted	102	34	33 (24-42)	0.0037	6333	427 (35-49048)	0.3118
	partially slatted	29	19	66 (48-83)		5069	880 (73-22961)	
Year	2019	70	29	41 (30-53)	0.9693	8844	3526 (46-49048)	0.0002
	2022	46	18	39 (25-53)		1230	110 (35-18920)	
	2023	15	6	40 (15-65)		5500	825 (37-19323)	
Months	November	41	16	39 (24-54)	0.9704	5500	825 (37-19323)	0.0038
	December	75	31	41 (30-52)		1380	110 (35-18920)	
	February	15	6	40 (15-65)		8276	2530 (37-49048)	

Inter-rated reliability was determined to analyze the degree of agreement between diagnostic methods using Cohen’s Kappa coefficient (Thrusfield 2007), using observations from the four farms tested with both methods. Results were interpreted as follows: < 0 = Less than chance agreement; 0.01–0.20 = light agreement; 0.21–0.40 = Fair agreement; 0.41–0.60 = Moderate agreement; 0.61–0.80 = Substantial agreement; 0.81–0.99 = Almost perfect agreement. Similarly, Spearman’s rank coefficient test was used to assess the correlation between OpG in samples analyzed with both methods. Statistical significance was considered for *p* < 0.05 for a double-sided test. All analyses were performed in the R statistical program (R Core team 2021).

## Results

### Estimated *C. suis* litter prevalence and relationship with explanatory variables

Oocysts were detected by Bailenger’s method in 53 of the 131 litters from the eight study farms, analyzed resulting in an overall estimated litter prevalence (95% CI) of infection of 40 (32–49)% (Table 1). The bivariate analysis of prevalence revealed significant differences between farms, and it was the highest in those with partially slatted dung floors. Earliest infection was detected in a 6-day-old litter medicated with oral TZ, in an 8-day-old litter treated with intramuscular TZ and in a 7-day-old litter not receiving any treatment. Prevalence increased with age with some differences depending on the litter treatment status; at 1 week of age it was the lowest in the non-medicated litters (8%), by 2 weeks of age it rose to 63%, 55% and 25% in non-medicated and orally and intramuscularly medicated litters, respectively, and at 3 weeks of age it was 80%, 73% and 55% in these groups, and prevalence was significantly lower in intramuscularly medicated compared to other litters (*p* < 0.05) (Table 1). Diarrhoeic, pasty and firm faeces were found in at least one pig in the litter in 25%, 25% and 50% of litters, respectively. The presence of diarrhoeic faeces in at least one pig in the litter was significantly higher in the non-medicated litters (40%) compared to those treated orally (18%) or intramuscularly (23%) (*p* < 0.05). Moreover, *C*. *suis* prevalence increased with decreasing faecal consistency: it was 31% in litters where all pigs had firm faeces, 39% in those with at least one pig with pasty faeces and firm faeces in other pigs and 61% in litters with at least one pig with diarrhoeic faces and firm or pasty faeces in other pigs (*p* < 0.05) (Table 1).

The multivariable logistic regression model confirmed the significant positive association between *C*. *suis* infection and increasing age, partially compared to fully slatted floors and diarrhoeic compared to firm faeces, and not with TZ treatments or year, and there was no remaining unexplained variation between farms (Table 2).

**Table 2:** Results from the logistic model analyzing the risk of *Cystoisospora suis* infection in piglets from eight farms, based on the presence of oocysts detected by Bailenger´s filtration-flotation concentration method

Parameters		Estimate	Std. error	*P* value
Intercept		3.02	0.73	<0.0001
Fixed variables	Level			
Age (weeks)	1	0		
	2	1.31	0.55	0.0175
	3	2.60	0.62	<0.0001
Type of dung floor area	completely slatted	0		
	partially slatted	1.90	0.86	0.0270
Faecal consistency	firm	0		
	pasty	0.79	0.56	0.1595
	diarrhoeic	1.56	0.55	0.0045
Preventive toltrazuril	injectable	0		
treatment	oral	0.12	0.84	0.8874
	not administered	0.93	0.68	0.1721
Year	2019	0		
	2022	0.01	0.62	0.9857
	2023	0.29	0.85	0.7305
Random variable	standard deviation			
Farm	0.00			

### Intensity of *C. suis* infection among positive litters and according to explanatory variables

Mean and median (range) OpG in Bailenger’s positive litters were 5880 and 623 (35-49048) OpG (Table 1). The median OpGs varied significantly between farms, months and years (*p* < 0.05) and marginally according to age (*p* < 0.10), being highest among 2-week-old litters in all treatment groups (Table 1).

In the multivariable analysis, Poisson regression revealed substantial overdispersion, and a negative binomial model was used. In this model, the number of oocysts/g of faeces in positive piglets was not significantly associated with any of the explanatory variables examined (*p* > 0.05, Table 3), although, numerically, it decreased with age and was lowest in litters with completely slatted floors and in those with at least one diarrhoeic pig, pigs treated with oral TZ and those investigated in the year 2022 (Table 1).

**Table 3:** Results from the negative binomial regression model analyzing *Cystoisospora suis* infection intensity in infected piglets from eight farms, based on Bailenger´s filtration-flotation concentration method

Parameters		estimate	Std. error	*P* value
Intercept		9.74	1.11	<0.0001
Fixed variables	Level			
Age (weeks)	1	0		
	2	-0.77	0.90	0.7803
	3	-2.01	0.77	0.8021
Type of dung floor area	completely slatted	0		
	partially slatted	2.36	0.92	0.2562
Faecal consistency	firm	0		
	pasty	0.32	0.80	0.8316
	liquid	-0.16	0.66	0.8523
Preventive toltrazuril	injectable	0		
treatment	oral	-1.95	1.03	0.3135
	not administered	0.43	0.67	0.6844
Year	2019	0		
	2022	-2.31	0.84	0.0944
	2023	0.57	1.08	0.6367
Random variable	standard deviation			
Farm	0.00			

### Performance and relationship between Bailenger’s and Joachim’s test results

The diagnostic test comparison Bailenger’s method was slower and more laborious compared to Joachim’s. Whilst both methods adequately removed a large part of fat present in the faeces samples allowing to visualize most oocysts clearly, the proportion of *C*. *suis* positives in the 61 samples from the four farms tested with both diagnostic methods was 39% with Bailenger’s and 15% with Joachim’s (*p* < 0.05). Test results coincided in 44 samples (8 positive and 36 negative), 16 samples were positive to Bailenger’s only and 1 was positive to Joachim’s only. The resulting Cohen’s Kappa coefficient (95% CI) was 0.34 (0.13–0.56) indicating only fair agreement. The median (range) OpG in the 8 positive samples were 953 (73-19323) oocysts for Bailenger’s and 4000 (1333-350684) oocysts for Joachim’s (*p* > 0.05), and Spearman’s rank correlation coefficient between the estimated number of OpG by both methods was rho = 0.47 and not statistically significant (*p* > 0.05).

## Discussion

The study provides evidence of a considerable *C*. *suis* infection in 1- to 3-week-old pig litter treated preventively when 48–72 h old with the anticoccidial TZ. Prevalence in litters treated orally was similar to that in untreated litters and much higher than in pigs treated by intramuscular injection. Infection was associated with diarrhoea in at least one animal in the litter and with farms not providing fully slatted floor in the farrowing units, which would favour the accumulation of faeces and infective oocysts and increase the risk of infection. Coccidiosis is a multifactorial disease and differences in management and hygiene practices, which are not always clear to identify, are responsible for large variations in the risk of *C*. *suis* infections between farms (Martineau and Castillo 2000; Aliaga-Leyton et al. 2011; Skampardonis et al. 2010; Hinney et al. 2020; Hinney et al. 2021). Clearly, examining a larger number of litters and including more non-medicated farms are required for a better understanding of oocyst excretion in untreated litters.

Diagnosis of infection was dependent on the method used and whilst Bailenger’s method detected a greater number of infected litters, the number of OpG in positives to both methods was numerically higher in Joachim’s method. The reasons for these paradoxical differences are not clear. In their study, Joachim et al. (2018a) compared the number of OpG detected with this method with those counted in smears by autofluorescence under UV light excitation and found a high correlation but higher counts with the latter method, probably due to oocysts being lost during sample processing or not floating well in the McMaster chamber. Therefore, they recommended autofluorescence for qualitative and semiquantitative assessment of oocysts in piglet faeces (Joachim et al. 2018a). Bailenger’s and Joachim’s methods are both flotation methods employing McMaster counting chambers, but they differ in several aspects, including the weight of the faeces sample processed (three times greater in Bailenger’s), the dilution of the sample (13.3 v/w for Bailenger and 71 v/w for Joachim’s) and Bailenger’s method — and not Joachim’s —, undergoing a pre-filtration step to remove the fat using acetoacetic buffer and ether also involving centrifugation and discarding the supernatant. The amount of faeces and sample dilution could crucially affect test sensitivity in samples with a low number of oocysts because oocyst concentration varies substantially within samples (Joachim et al. 2018a). Joachim et al.’s (2018a) method was prepared to examine individual piglet faecal examples and not pooled samples. Clearly, a more robust assessment of the diagnostic validity of the two methods should be carried out by preparing oocyst spiked samples with comparable faecal amounts using different faecal consistencies.

The dynamics of oocysts excretion determines the level of environmental contamination and conditions the risk of infection and is influenced by the administration of TZ. In this study prevalence gradually increased until 3 weeks of age in treated and untreated litters, whilst infection intensity was highest in 1- and 2-week-old litters. This pattern is similar to that reported in a previous study of natural infection in non-medicated piglets (Sotiraki et al. 2008). An increasing risk of infection would be associated with longer exposure to and accumulation of sporulated oocysts in the pen, and lower infection intensity in the oldest pigs suggests they developed acquired immunity, restricting *C*. *suis* multiplication in intestinal cells. In contrast, in other studies in TZ-treated piglets, oocyst excretion was very low or absent during the first 3 weeks of life (Bach et al. 2003; Kreiner et al. 2011; Hiob et al. 2019). Experimental pharmacokinetic studies of TZ and its active metabolite TZ-SO_2_ indicated peak concentrations of these compounds in jejunum and ileum, 24 h and 5 days post-treatment for TZ and on day 13 for TZ-SO_2_, and pigs treated intramuscularly achieved greater and more sustained levels compared to those treated orally (Karembe et al. 2021). The finding in the present study of a high number of oocysts in 1- to 2-week-old piglets treated litters followed by an increasing age-trend in the prevalence of infection — albeit lower in injected litters — suggests some animals in the litter were not effectively treated or oral treatments were regurgitated, which is a recognized problem (Hiob et al. 2019), or that the treatment’s residual activity was lower than expected, possibly due to underdosing or other unknown reasons. Other studies in Europe have reported substantial oocysts excretion in TZ-treated piglets and lack of an association between TZ administration and litter *C*. *suis* positivity rates (Hinney et al. 2020 and 2021; Nunes et al. 2023). These findings also inevitably raise the question of possible tolerance or resistance of *C*. *suis* strains to TZ in these farms, as has been reported elsewhere (Shrestha et al. 2017). This could be assessed experimentally as described by Shrestha et al. (2017), and under natural conditions in study farms by including non-medicated control groups or following a longitudinal study design removing treatment from subsequent litters, but this was not an option in the present study.

The low prevalence of infection in non-medicated 1-week-old litters in farm 5 followed by a gradual increase, similar to that in orally treated litters from other farms, suggests that adequate control of coccidiosis can be achieved without resorting to preventive anticoccidial treatments. However, the proportion of litters with at least one diarrhoeic piglet was significantly greater in the non-medicated farm than in treated farms. Whilst the presence of *C*. *suis* in faecal samples can be associated with diarrhoea (Joachim and Shrestha 2020; Nunes et al. 2023), a better assessment of the impact of cystoisosporosis in piglets requires investigating weight gain rates as well as other neonatal coinfections (Joachim et al. 2018b).

In contrast to oocyst presence, infection intensity was not significantly associated with faecal consistency. Studies of the relationship between the number of OpG and faecal consistency show contradictory results, and this is partly because diarrhoea caused by *C*. *suis* and oocysts excretion are not always synchronous (Mundt et al. 2006), and neonatal diarrhoea in commercially raised pigs is a syndrome caused by several protozoa, bacteria and virus species which do not necessarily occur simultaneously (Ruiz et al. 2016). Also, Mundt et al. (2006) argued that a lower number of OpG in diarrhoeic samples could be the result of (i) a dilution effect because diarrhoeic pigs produce a larger volume of faeces and (ii) rapid enterocyte turnover caused by inflammation limiting the parasite’s chances of completing their intracellular development and consequently oocyst production. Moreover, experimental infection studies with *Eimeria* spp. coccidia in chickens and sheep demonstrated ‘crowding’ density-dependent processes whereby as the infective dose is increased more oocysts are excreted until an excessive dose is reached that results in lower oocysts output (Pout 1965; Lozte and Leek 1970; Fernando 1990). This was considered the result of host responses or caused by habitat limitation.

The prospects of controlling parasite infections by permanent administration of preventive medication are challenged by the emergence of drug-resistant parasites. A recommended approach to slow the development of widespread anthelmintic resistance is maintaining a refugium of untreated hosts and environments for drug-sensitive parasites thereby preventing exclusive colonization of resistant strains (Hodgkinson et al. 2019). A possible application of this concept for the control of neonatal coccidiosis in piglets could be to alternate periods using preventive treatments with other comparatively long periods with no medication. The success of such an approach is however questionable given that coccidia have a much faster development and multiplication rate compared to helminths, and neonatal pigs lack acquired immunity against the parasite. In any case, this strategy must be accompanied with high husbandry and sanitary standards to prevent progressive oocyst accumulation in the environment, including the use of appropriate disinfectants (Straberg and Daugschies 2007; Hinney et al. 2021).

Preventive toltrazuril treatments for the control of neonatal coccidiosis in pigs may be widely used in Murcia Region, and whilst it appears to prevent disease outbreaks, a relatively large proportion of piglets may become infected and eliminate a high number of oocysts into the environment. This may be associated with treatment administration failures, reduced toltrazuril residual efficacy or low susceptibility of *C. suis* strains in study farms. The comparable pattern of infection in non-medicated and some medicated litters indicates that coccidiosis can be to some extent prevented through exemplary management. We support evidence that Bailenger’s and Joachim’s flotation concentration methods allow removing a large part of the fat present in the faeces samples and visualizing most oocysts clearly. Still, Bailenger’s greater ability to detect oocysts in pooled faecal samples would favour this method in studies to estimate the prevalence of infection using such samples as here described.

### Supplementary information

ESM 1 (MP4 13919 kb)

ESM 2 (MP4 17228 kb)

## Data Availability

Data supporting this study may be made available upon request to the corresponding author.
